# Independent risk factors and outcomes for ventilator-associated pneumonia due to multidrug-resistant organisms after cardiac valvular surgery

**DOI:** 10.3389/fcvm.2025.1570195

**Published:** 2025-04-03

**Authors:** XiaoLiang Chen, LaiYha Yan, ShunYing Zhao, XiaoYan Hu, GuoFeng Shao, Ni Li, LinWen Zhu

**Affiliations:** ^1^ Department of Cardiosurgery Intensive Care Unit, Ningbo Medical Centre Lihuili Hospital, Ningbo University, Ningbo, China; ^2^ Department of Cardiothoracic Surgery, Ningbo Medical Centre Lihuili Hospital, Ningbo University, Ningbo, China

**Keywords:** cardiac valvular surgery, multidrug-resistant organisms, risk factors, ventilator-associated pneumonia, valvular heart disease

## Abstract

**Background:**

Although numerous studies have documented the risk factors for ventilator-associated pneumonia (VAP) after cardiac surgery, most of these studies included heterogeneous patient populations. This study aimed to explore the risk factors for VAP caused by multidrug-resistant organisms (MDRO) in patients admitted to the cardiosurgery intensive care unit (CSICU) following cardiac valvular surgery.

**Methods:**

This was a single-center, retrospective study. The clinical data of adult VAP patients following cardiac valvular surgery from January 2021 to December 2023 were analyzed. Patients were divided into MDRO VAP and non-MDRO VAP groups. Perioperative clinical data and postoperative follow-up results were collected for both groups. Univariable and multivariable logistic regression analyses were performed to identify risk factors for MDRO VAP, and the outcomes of MDRO VAP patients were analyzed. The species of pathogens isolated from the VAP patients were also investigated.

**Results:**

A total of 109 VAP patients were included in this study, including 47 patients with MDRO VAP and 62 patients with non-MDRO VAP. Multivariable logistic regression analysis identified that independent risk factors for MDRO VAP included preoperative hypoalbuminemia (OR, 0.838; CI, 0.733–0.957; *p* = 0.009), prolonged mechanical ventilation (OR, 1.173; CI, 1.005–1.369; *p* = 0.043), and extended broad-spectrum antibiotic therapy (OR, 1.112; CI, 1.019–1.213; *p* = 0.018). Patients with MDRO VAP had significantly longer ICU stays, total hospital stays, and higher hospitalization costs than non-MDRO VAP patients. The in-hospital mortality rate of the MDRO VAP group was significantly higher than that of the non-MDRO VAP group (29.79% vs. 1.61%, *p* < 0.001). Gram-negative bacilli were the predominant pathogens in MDRO VAP patients (97.87%), with the highest rate of *Pseudomonas aeruginosa* (29.79%).

**Conclusion:**

Postoperative MDRO VAP in patients undergoing cardiac valvular surgery is linked to severe clinical outcomes. Greater attention should be given to patients with prolonged mechanical ventilation, extended broad-spectrum antibiotic therapy, and preoperative hypoalbuminemia to prevent MDRO VAP infections.

## 1 Introduction

Valvular heart disease (VHD) is a type of cardiovascular disease characterized by structural and/or functional abnormalities of the heart valves. The epidemiology of VHD varies significantly worldwide. In high-income countries, functional and degenerative diseases are more prevalent, whereas rheumatic heart disease is common in low- and middle-income countries (1). It is a leading cause of cardiovascular morbidity and mortality globally, affecting approximately 41 million individuals (2).

Cardiac valvular surgery is a crucial treatment for valvular heart disease, effectively improving patients' condition, alleviating clinical symptoms, and enhancing their quality of life (3). However, Cardiac valvular surgery has the characteristics of significant trauma, multiple interventional procedures, and complex operations. Additionally, patients undergoing cardiac valve surgery are predominantly middle-aged and elderly individuals, often with underlying comorbidities, making them a high-risk group for ventilator-associated pneumonia (VAP) (4). The occurrence of VAP in cardiac surgery patients leads to increased mortality and prolonged hospital stays, imposing a significant financial burden on patients and their families (5, 6).

The rise and widespread prevalence of multidrug-resistant organisms (MDRO) pose a significant threat to human health. Multidrug-resistant organisms are increasingly prevalent and are associated with significant morbidity and mortality rates. A study on clinical characteristics and risk factors for multidrug-resistant(MDR) bacteria following adult cardiac surgery revealed that the incidence of lower respiratory tract infections was 29.0%, MDRO infections 8.6%, and all-cause mortality was significantly higher in MDRO patients (7). A single-center retrospective study of adult patients following cardiac surgery found that gram-negative bacteria(GNB) was the most common MDR pathogen in ventilator-associated pneumonia, with the incidence of multidrug-resistant bacteria reaching 55.0%. Specifically, patients who develop MDRO-related VAP after cardiac surgery often experience prolonged mechanical ventilation, extended intensive care unit (ICU) stay, and increased healthcare costs, all of which can severely impact their prognosis (8). Various clinical studies indicate that the prevalence of MDRO infections varies across regions and hospitals. However, the overall impact of MDRO infections remains consistent. Bacterial drug resistance is a significant concern, as the emergence and spread of multidrug-resistant bacteria pose major challenges to effective clinical infection management (7).

However, the risk factors for MDRO-induced VAP after valvular heart surgery in adults have not been adequately reported. Therefore, it is essential to investigate the risk factors for MDRO VAP following cardiac valvular surgery to develop effective prevention and treatment strategies. We conducted a retrospective study to identify potential risk factors and outcomes of MDRO infections in patients undergoing cardiac valvular surgery at our institution from January 2021 to December 2023.

## 2 Materials and methods

### 2.1 Design and patients

This was a retrospective case-control study. Data was collected from the electronic medical records of patients who were admitted to the CSICU after valvular heart surgery between January 2021 and December 2023. During this period, 763 patients underwent ventilator-assisted breathing after cardiac valve surgery, and 109 of them developed ventilator-associated pneumonia (VAP). Then, 109 patients were categorized into the MDRO VAP group (47 cases) or non-MDRO VAP group (62 cases).

Ventilator-associated pneumonia was defined as pneumonia occurring >48 h after endotracheal intubation according to the new guidelines of the American Thoracic Society and the Infectious Diseases Society of America (9, 10). Confirmed VAP was based on clinical and microbiological criteria. Clinical criteria were the presence of new or progressive infiltrates on chest x-ray images and accompanied by two or more of the following: (1) Fever (>38 °C) or hypothermia (<36 °C). (2) White blood cell (WBC) count >10 × 109/L or <4 × 109/L. (3) Purulent secretions in the trachea and bronchus. Microbiological criteria were the secretions extracted by endotracheal aspiration (ETA) which were considered positive for a total colony count of ≥10^5^ cfu/ml; sometimes samples taken by bronchoalveolar lavage (BAL) are considered positive for a total colony count of ≥10^4^ cfu/ml.

The inclusion criteria were as follows: (1) age ≥18 years. (2) mechanical ventilation >48 h. (3) meeting the VAP diagnostic criteria, (4) cardiac valvular surgery using cardiopulmonary bypass, and (5) no other surgery performed during hospitalization. The exclusion criteria were: (1) pre-existing lung infection on admission, (2) patients who died within 48 h after surgery, (3) minimally invasive procedures, such as thoracoscopy or transcatheter aortic valve implantation (TAVI), and (4) combination with other surgeries (e.g., coronary artery bypass grafting combined with cardiac valvular surgery). All surgeries were performed by the same senior surgeon supported by the same medical team.

### 2.2 Data collection

Data were collected from the electronic medical record system of patients who underwent cardiac valvular surgery between January 2021 and December 2023. The preoperative laboratory data were recorded from admission to the last examination before surgery. Postoperative laboratory data were recorded based on the following criteria: The partial pressure of oxygen in arterial blood/fraction of inspired oxygen (PaO2/FiO2) ratio refers to the mean value within 48 h of mechanical ventilation following cardiac surgery. High-sensitivity cardiac troponin (hs-cTn) is the highest rise value recorded within 72 h after cardiac surgery. Postoperative creatinine is the highest value recorded within 48 h after cardiac surgery.

Preoperative data collected for the study included demographics, comorbidities, and laboratory results, including age, sex, body mass index(BMI), hypertension, diabetes mellitus, creatinine clearance, hematocrit, serum albumin, pulmonary hypertension, atrial fibrillation, prior cardiac valvular surgery, and the New York Heart Association (NYHA) classification. Operative data included the type of surgery, surgical access route, intraoperative cardiopulmonary bypass time, aortic cross-clamp time, and the use of blood products during surgery.

Postoperative data included creatinine, PaO2/FiO2 ratio, high-sensitivity cardiac troponin(hs-cTn), mechanical ventilation time, ICU stay time, secondary intubation, tracheotomy, re-admission to the ICU, duration of broad-spectrum antibiotic therapy, and postoperative complications. Postoperative complications included acute respiratory distress syndrome (ARDS), perioperative myocardial infarction (PMI), low cardiac output syndrome (LCOS), and acute kidney injury (AKI).

Additionally, data on extracorporeal membrane oxygenation (ECMO) support, intra-aortic balloon pump (IABP) support, continuous renal replacement therapy (CRRT), total hospital stay time, total hospitalization costs, and the time of death after discharge were recorded. Microbiological data and the antibiotic therapies prescribed were also recorded. Multi-drug resistance was defined as non-susceptibility to at least one agent in three or more antimicrobial categories (11).

The diagnostic criteria for AKI include an increase in the absolute value of serum creatinine by 0.3 mg/dl (26.5 µmol/L) or a urine output <0.5 ml/ (kg. h) for more than 6 h within 48 h after cardiac surgery (12). The diagnostic criteria for PMI is based on the latest European Association of Cardio-Thoracic Surgery (EACTS) expert consensus statement on PMI after cardiac surgery in 2024 (13). Acute respiratory distress syndrome (ARDS) is characterized by acute hypoxaemic respiratory failure with bilateral infiltrates on chest imaging, which is not fully explained by cardiac failure or fluid overload. ARDS is defined by the Berlin criteria (14). Postoperative LCOS is defined as the need for inotropes and/or mechanical circulatory assistance for at least 24 h postoperatively during the first five postoperative days (15).

### 2.3 Bacterial isolates and antimicrobial susceptibility testing

Deep sputum samples from the lower respiratory tract were collected for pathogen culture and microscopy using either endotracheal aspiration (ETA) or bronchoalveolar lavage (BAL), with BAL accounting for 41.28% of the samples. Subsequently, the specimens were inoculated into the corresponding media and identified using the VITEK MS microbial mass spectrometer (BioMérieux, France) according to the fourth edition of the National Clinical Laboratory Procedures. Quality control strains, including *Staphylococcus aureus* (ATCC 25923), *Klebsiella pneumoniae* (ATCC 700603), *Escherichia coli* (ATCC 25922), and *Pseudomonas aeruginosa* (ATCC 27853), were obtained from the American Type Culture Collection Global Bioresource Center (Manassas, VA, USA).

Antimicrobial susceptibility test was carried out using the VITEK-2 COMPACT automatic microbial identification system to determine the minimum inhibitory concentration(MIC) value. Supplementary testing was performed using the modified Kirby-Bauer disc diffusion method. Results were interpreted based on the standards outlined in the Clinical and Laboratory Standards Institute (CLSI) M100-Ed33 (16).

Due to *Stenotrophomonas*' intrinsic resistance to carbapenems, we omitted imipenem/meropenem from the antimicrobial susceptibility test for *Stenotrophomonas* in this study. The antibiogram included minocycline, trimethoprim-sulfamethoxazole, levofloxacin, and ceftazidime. In this study, we found 6 VAP patients with *Stenotrophomonas* infection who were sensitive to all four drugs in the antibiogram. Clinicians had no difficulty selecting antibiotics due to the natural MDRO profile and absence of related complications (17). Therefore, in this study, we included *Stenotrophomonas*-infected VAP patients in the non-MDRO VAP group.

### 2.4 Statistical analyses

For continuous variables, the Kolmogorov–Smirnov test was used to assess normal distribution. Continuous variables with a normal distribution are expressed as mean ± standard deviation (SD) and compared using Student's *t*-test; otherwise, they are expressed as median (Q25, Q75) and compared using the Mann–Whitney *U* test. Categorical data are shown as numbers (%) and analyzed using the chi-square test or Fisher's exact test as appropriate. *p*-value <0.05 was considered statistically significant. Univariate and multivariate regression analyses were performed to identify the risk factors for VAP. The odds ratio (OR) with a 95% confidence interval (CI) was used to quantify the strength of associations between variables. Univariate logistic regression analysis was conducted to assess the independent influence of each variable. Baseline variables with a *p* value <0.05 in univariate analysis were included in the multivariate analysis. A two-tailed *p*-value <0.05 was considered statistically significant. The Kaplan–Meier method was used to analyze 60-day survival rates, and the log-rank test was used to generate *p*-values. All statistical analyses were performed using SPSS Statistics 26.0 (IBM Corp., Armonk, NY).

## 3 Results

**Figure 1:**
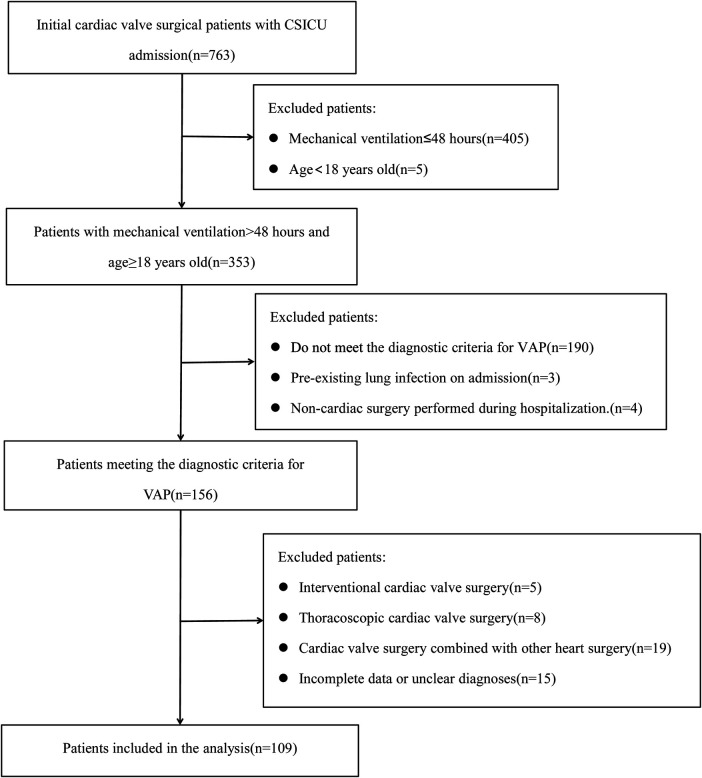
Flowchart for selecting ventilator-associated pneumonia after cardiac valvular surgery. CSICU, cardiosurgery intensive care unit; VAP, ventilator-associated pneumonia.

Between January 2021 and December 2023, a total of 763 patients requiring mechanical ventilation were admitted to the CSICU following cardiac valve surgery. After applying exclusion criteria, 109 patients who underwent cardiac surgery with cardiopulmonary bypass (CPB) and met the study criteria were identified, as illustrated in Figure 1. The preoperative, operative, and postoperative clinical data of the MDRO VAP and non-MDRO VAP groups were statistically analyzed. Cardiac surgical procedures included mitral valve replacement(MVR) in 26 (23.85%), aortic valve replacement(AVR) in 15 (13.76%), combined mitral and tricuspid valve surgery in 26 (23.85%), combined aortic and mitral valve replacement in 12 (11.01%), tricuspid valve surgery in 13 (11.93%), and others in 17 (15.60%).

### 3.1 Risk factors for MDRO VAP infection in patients after cardiac valvular surgery

In the 109 patients with VAP, there were no significant differences in baseline characteristics between the MDRO and non-MDRO groups, except for preoperative albumin levels (*p* = 0.005), C-reactive protein-to-albumin ratio (CAR) (*p* = 0.037), and creatinine levels (*p* = 0.026) (Table 1).

**Table 1:** Comparison of the preoperative data between patients with and without MDRO VAP.

Valuables	MDRO VAP (*n* = 47)	Non-MDRO VAP (*n* = 62)	*p* Value
Age, years	64.30 ± 10.43	64.59 ± 11.85	0.893
Female, *n* (%)	23 (48.94%)	31 (50.00%)	0.912
BMI (kg/m^2^)	23.18 ± 3.76	22.29 ± 3.59	0.204
CAR	0.05 (0.02, 0.33)	0.02 (0.01, 0.14)	**0** **.** **037**
C-reactive protein (mg/L)	0.82 (1.67, 10.98)	0.50 (0.72, 5.48)	0.064
Hemoglobin (g/L)	11.88 ± 2.23	12.44 ± 2.11	0.183
Albumin (g/L)	36.27 ± 4.84	38.64 ± 3.84	**0**.**005**
Hematocrit (%)	36.45 ± 6.32	37.24 ± 5.96	0.505
Creatinine (μmol/L)	78.00 (60.00, 122.50)	67.50 (56.50, 91.25)	**0**.**026**
NYHA class III–IV, *n* (%)	41 (87.23%)	53 (85.48%)	0.793
Hypertension, *n* (%)	17 (36.17%)	24 (38.71%)	0.786
Atrial fibrillation, *n* (%)	22 (46.81%)	21 (33.87%)	0.171
Diabetes mellitus, *n* (%)	6 (12.77%)	2 (3.23%)	0.128
Pulmonary hypertension, *n* (%)	11 (23.40%)	10 (16.13%)	0.340
Prior operative cardiac surgery, *n* (%)	15 (31.92%)	10 (16.13%)	0.052
Surgical access route			
Full sternotomy, *n* (%)	38 (80.85%)	52 (83.87%)	0.681
Partial upper sternotomy, *n* (%)	2 (4.26%)	4 (6.45%)	0.941
Right anterolateral thoracotomy, *n* (%)	7 (14.89%)	6 (9.68%)	0.405
The type of operation			
Mitral valve replacement, *n* (%)	10 (21.28%)	16 (25.81%)	0.583
Aortic valve replacement, *n* (%)	5 (10.64%)	10 (16.13%)	0.410
Concomitant aortic and valve replacement, *n* (%)	5 (10.64%)	7 (11.29%)	0.914
Combined mitral and tricuspid valve surgery, *n* (%)	11 (23.40%)	12 (19.36%)	0.608

The bold *p* values indicate a statistically significant difference between the two groups.

BMI, body mass index; NYHA, New York heart association; CAR, C-reactive protein-to-albumin ratio.

In addition, we further analyzed the risk factors for MDRO VAP infection in patients after cardiac valvular surgery. Univariate logistic regression analyses were performed to identify basic demographic characteristics, clinical characteristics, and laboratory parameters. Univariate logistic regression analysis identified preoperative albumin (OR, 0.878; CI, 0.798–0.966; *p* *=* 0.008), aortic cross-clamp time (OR, 1.014; CI, 1.002–1.026; *p* *=* 0.025), mechanical ventilation time (OR, 1.272; CI, 1.140–1.421; *p* < 0.001), postoperative ICU stay time (OR, 1.140; CI, 1.069–1.215; *p* < 0.001), tracheotomy (OR, 14.062; CI, 3.025–65.368; *p* *=* 0.001), total hospital stay time (OR, 1.061; CI, 1.028–1.096; *p* < 0.001), re-intubation (OR, 3.919; CI, 1.145–13.417; *p* *=* 0.03), duration of VAP broad-spectrum antibiotic therapy (OR, 1.093; CI, 1.049–1.139; *p* < 0.001), low cardiac output syndrome (OR, 8.108; CI, 1.683–39.070; *p* *=* 0.009), perioperative myocardial infarction (OR, 2.322; CI, 1.034–5.214; *p* *=* 0.041), and CRRT support (OR, 12.207; CI, 3.325–44.816; *p* < 0.001) as risk factors at a level of statistical significance for MDRO VAP (Table 2). Moreover, a multivariate regression analysis was performed. The results indicated that preoperative albumin (OR, 0.838; CI, 0.733–0.957; *p* *=* 0.009), mechanical ventilation time (OR, 1.173; CI, 1.005–1.369; *p* *=* 0.043), and duration of VAP broad-spectrum antibiotic therapy (OR, 1.112; CI, 1.019–1.213; *p* *=* 0.018) were independent risk factors for MDRO VAP (Table 3).

**Table 2:** Univariate logistic regression analyses of risk factors for the patient with MDRO VAP.

Variable	Odds ratio	95% confidence interval	*p* value
Preoperative related factors			
BMI (kg/m^2^)	1.070	0.964–1.189	0.203
CAR	1.190	0.733–1.932	0.482
C-reactive protein (mg/L)	1.002	0.988–1.016	0.768
Hemoglobin (g/L)	0.886	0.742–1.059	0.183
Albumin (g/L)	0.878	0.798–0.966	0.008
Hematocrit (%)	0.979	0.919–1.042	0.501
Creatinine (μmol/L)	1.004	0.998–1.010	0.172
Hypertension	0.897	0.410–1.966	0.786
Atrial fibrillation	1.718	0.789–3.740	0.173
Diabetes mellitus	4.390	0.844–22.834	0.079
Pulmonary hypertension	1.589	0.611–4.133	0.342
Prior operative cardiac surgery	2.498	0.973–6.418	0.057
Intraoperative related factors			
Intraoperative blood transfusion (ml)	1.000	1.000–1.001	0.215
Aortic cross-clamp time	1.014	1.002–1.026	0.025
Cardiopulmonary bypass time	1.008	1.000–1.016	0.058
Postoperative related factors			
Mechanical ventilation time	1.272	1.140–1.421	<0.001
ICU stay time	1.140	1.069–1.215	<0.001
Total hospital stay time	1.061	1.028–1.096	<0.001
Re-intubation	3.919	1.145–13.417	0.03
Tracheotomy	14.062	3.025–65.368	0.001
Re-admission to ICU	3.442	0.840–14.108	0.086
Postoperative creatinine (μmol/L)	1.004	0.998–1.010	0.223
PaO2/FiO2 (mmHg)	0.993	0.987–1.000	0.58
Duration of VAP broad-spectrum antibiotic therapy	1.093	1.049–1.139	<0.001
High-sensitivity cardiac troponin	1.023	0.985–1.061	0.243
Postoperative complications			
Acute respiratory distress syndrome	2.523	0.845–7.533	0.097
Acute renal injury	1.943	0.837–4.508	0.122
Low cardiac output syndrome	8.108	1.683–39.070	0.009
Perioperative myocardial infarction	2.322	1.034–5.214	0.041
Postoperative mechanical support			
VA-ECMO	4.159	0.419–41.323	0.224
CRRT	12.207	3.325–44.816	<0.001

BMI, body mass index; ICU, intensive care unit; PaO2/FiO2, partial pressure of oxygen in arterial blood/fraction of inspired oxygen; CRRT, continuous renal replacement therapy; VA-ECMO, veno-arterial extracorporeal membrane oxygenation, CAR, C-reactive protein-to-albumin ratio.

**Table 3:** Multivariable logistic regression analyses of risk factors for the patient with MDRO VAP.

Variable	Odds ratio	95% confidence interval	*p* value
Preoperative albumin	0.838	0.733–0.957	0.009
Aortic cross-clamp time	1.009	0.990–1.029	0.35
Mechanical ventilation time	1.173	1.005–1.369	0.043
ICU stay time	1.163	0.952–1.420	0.139
Total hospital stay time	0.908	0.830–0.993	0.35
Re-intubation	1.195	0.169–8.449	0.859
Tracheotomy	0.056	0.002 = 1.772	0.102
Duration of VAP broad-spectrum antibiotic therapy	1.112	1.019–1.213	0.018
Low cardiac output syndrome	1.046	1.330–8.253	0.966
Perioperative myocardial infarction	0.954	0.270–3.373	0.941
CRRT	1.570	0.246–10.034	0.633

ICU, intensive care unit; CRRT, Continuous renal replacement therapy.

### 3.2 Prognosis of patients with MDRO VAP after cardiac valvular surgery

We analyzed the factors affecting the prognosis of patients with MDRO VAP after cardiac valvular surgery. Results showed that postoperative ICU stay time [14.00(7.00, 31.00) d vs. 5.00(4.00, 6.25) d, *p* < 0.001], and total hospital stay time (43.53 ± 28.95d vs. 25.63 ± 10.80d, *p* < 0.001) were significantly longer. The total cost of hospitalization (44,896.14 ± 37,322.26$ vs. 20,280.30 ± 8,511.71$, *p* < 0.001) was also significantly higher in the MDRO VAP group than in the non-MDRO VAP group. The in-hospital mortality rate was significantly higher in patients with MDRO VAP (29.79% vs. 1.61%, *p* < 0.001) (Table 4).

**Table 4:** Comparison of the postoperative outcomes of MDRO VAP and non-MDRO VAP patients.

Valuables	MDRO VAP (*n* = 47)	Non-MDRO VAP (*n* = 62)	*p* value
Mechanical ventilation time (day)	10.23 ± 7.23	3.73 ± 3.52	<0.001
ICU stay time (day)	14.00 (7.00, 31.00)	5.00 (4.00, 6.25)	<0.001
Total hospital stay time (day)	43.53 ± 28.95	25.63 ± 10.80	<0.001
total cost of hospitalization (US$)	44,896.14 ± 37,322.26	20,280.30 ± 8,511.71	<0.001
In-hospital mortality, *n* (%)	14 (29.79%)	1 (1.61%)	<0.001

ICU, intensive care unit.

### 3.3 Kaplan–meier survival analysis

**Figure 2:**
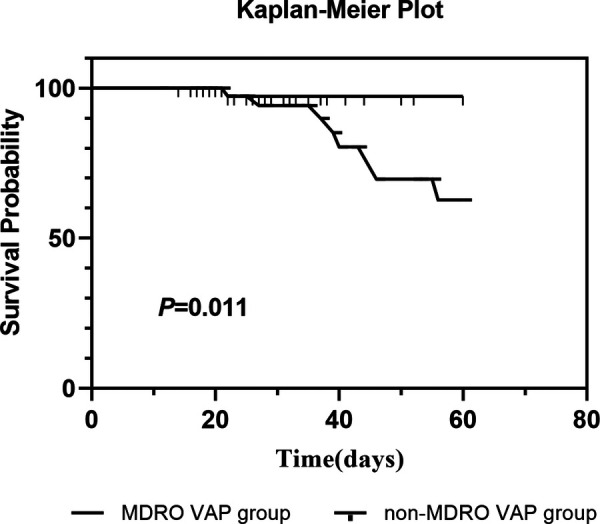
Probability of survival through day 60 in patients with and without MDRO ventilator associated pneumonia.

In the Kaplan–Meier survival analysis, patients in the MDRO VAP group following cardiac valvular surgery showed a significantly lower 60-day survival probability compared to those in the non-MDRO VAP group (*p* = 0.011) (Figure 2).

### 3.4 Analysis of pathogens in MDRO VAP patients after cardiac valvular surgery

The organisms recovered from the VAP patients' specimens were identified. In the MDRO VAP group, Gram-negative bacteria were most commonly found (97.87%), mainly *Pseudomonas aeruginosa* (*n* = 14, 29.79%), *Acinetobacter baumannii* (*n* = 10, 21.28%), *Klebsiella pneumoniae* (*n* = 9, 19.15%), *Enterobacter cloacae* (*n* = 4, 8.51%), and *Escherichia coli* (*n* = 4, 8.51%). However, the Gram-positive bacteria that accounted for 2.13% were *Staphylococcus aureus* (*n* = 1, 2.13%) (Table 5).

**Table 5:** Organisms isolated from 109 patients with ventilator-associated pneumonia.

Organisms	MDRO VAP (*n* = 47)	Non-MDRO VAP (*n* = 62)
Gram-positive bacteria		
*Staphylococcus aureus*	1 (2.13%)	4 (6.45%)
Gram-negative bacteria		
*Acinetobacter baumannii*	10 (21.28%)	8 (12.90%)
*Klebsiella pneumoniae*	9 (19.15%)	27 (43.55%)
*Pseudomonas aeruginosa*	14 (29.79%)	4 (6.45%)
*Enterobacter cloacae*	4 (8.51%)	3 (4.84%)
*Escherichia coli*	4 (8.51%)	0 (0.00%)
*Stenotrophomonas maltophilia*	0 (0.00%)	6 (9.68%)
Others	5 (10.63%)	10 (16.13%)

## 4 Discussion

Ventilator-associated pneumonia is one of the most common hospital-acquired infections in patients who have undergone cardiac surgery (8). Research has shown that the incidence of VAP after cardiac procedures ranges from 2.1% to 13%, with an occurrence rate of 17.1 to 34.5 cases per 1,000 ventilator days (18).

Patients with valvular heart disease often experience a prolonged disease course, which may lead to complications such as heart failure, pulmonary hypertension, and hypoalbuminemia (19). Additionally, cardiac valvular surgery typically involves hypothermic anesthesia and cardiopulmonary bypass, both of which can contribute to the development of VAP (20). However, the types of multidrug-resistant strains that cause VAP can vary significantly between hospitals and among patient populations, particularly in relation to existing comorbidities (21). Therefore, it is crucial to thoroughly assess the risk factors for MDRO-associated VAP following valvular heart surgery.

In our MDRO VAP population, Gram-negative bacteria were the predominant pathogens (97.87%). Notably, resistant pathogens (including carbapenem-resistant *Pseudomonas aeruginosa*, carbapenem-resistant *Acinetobacter baumannii*, and *Klebsiella pneumoniae*) accounted for 70.22% of the Gram-negative bacteria among MDRO VAP pathogens in our study. In addition, polymicrobial infection was observed in 30.28% of VAP patients. This high prevalence of resistant and polymicrobial infections highlighted the complexity of VAP pathogens after cardiac valvular surgery.

Valvular heart disease is common in patients with impaired renal function (22). Our study found that preoperative creatinine levels were higher in MDRO VAP patients than in non-MDRO VAP patients. Liao et al. found that preoperative renal insufficiency was associated with increased rates of ventilator use exceeding 48 h, readmission, reoperation, and prolonged hospital stays, as well as an elevated risk of infectious events, including pneumonia and septic shock (23). Preoperative renal insufficiency in cardiac valve surgery patients may contribute to postoperative MDRO VAP. Our study highlighted the independent risk factors of MDRO VAP in cardiac valvular surgical patients in the CSICU. Patients were more prone to develop MDRO VAP if they experienced prolonged mechanical ventilation, prolonged broad-spectrum antibiotic therapy, or preoperative hypoalbuminemia.

Wang and colleagues conducted a retrospective study and identified the risk factors for MDRO VAP after cardiac surgery. They found that independent risk factors for MDRO VAP included a preoperative creatinine clearance rate ≥86.6 ml, intraoperative cardiopulmonary bypass time ≥151 min, and postoperative acute kidney injury (8). However, our research findings differ from the previous study, possibly because our study subjects were exclusively patients undergoing heart valve surgery. In contrast, Wang et al.'s subjects included patients undergoing heart valve, coronary graft, aorta, and congenital heart disease surgeries, with pure heart valve surgery accounting for only 34.4%.

Prolonged mechanical ventilation is a common complication following cardiac surgery, as it increases the risk of respiratory muscle weakness and pulmonary infections. Additionally, it has been associated with a reduced postoperative quality of life, higher rates of intensive care unit readmissions, multiple postoperative complications, and an increased risk of mortality (24). Our study found that patients who underwent cardiac valve surgery and required prolonged mechanical ventilation were more likely to develop MDRO VAP. In cardiac surgery, early extubation is a widely discussed topic. However, the risk of morbidity associated with reintubation remains a concern for early extubation strategies. In a retrospective cohort study involving over 6,600 patients who underwent isolated coronary artery bypass graft (CABG) surgery and 2,900 patients who underwent isolated aortic valve replacement (AVR) surgery, the reintubation rates during the first 30 postoperative days were 1.7% for CABG and 1.5% for AVR. The study indicated that early extubation did not increase the risk of reintubation. Additionally, the researchers found that advanced age was associated with a higher risk of reintubation after elective cardiac surgery (25). Substantial evidence has demonstrated that early tracheal extubation is associated with a shorter ICU length of stay without an increase in the rate of postoperative complications in patients who do not have multiple comorbidities, are elderly, or have undergone complex non-elective procedures after cardiac surgery (26). Delayed extubation after cardiac surgery may result in more postoperative complications (e.g., pneumonia, cardiac arrest, reintubation, and other complications) and a higher risk of death. Therefore, clinicians should consider early extubation for low-risk patients after cardiac surgery. For medium-risk and high-risk patients, clinicians can assess extubation timing and develop specific treatment strategies as quickly as possible with the help of risk stratification using the prediction scoring model (27).

Antibacterial drugs can inhibit or kill bacteria and have become a powerful weapon in the treatment of bacterial infections. However, the role of antibacterial drugs is dual in nature, encompassing both therapeutic effects and adverse reactions. If antibiotics, particularly broad-spectrum antibiotics, are not used appropriately, such as when their administration is prolonged, it will lead to the occurrence of multi-drug resistant bacteria (28, 29). Given the high morbidity and mortality rates associated with MDRO VAP, our study aimed to evaluate the duration of broad-spectrum antibiotic therapy in patients with VAP after cardiac valve surgery to optimize the use of antibiotics in patients with VAP infection after cardiac valve surgery. Patients in the CSICU who are critically ill often face multiple bacterial infections, especially those with VAP. Consequently, they frequently receive prolonged broad-spectrum antibiotic therapy, which can contribute to the development of resistant bacterial infections in the lungs following cardiac valvular surgery. In a retrospective observational study examining the temporal association between carbapenem usage and antimicrobial resistance among major Gram-negative bacteria, researchers observed that carbapenem consumption, as well as resistance to imipenem in *Acinetobacter baumannii* and *Pseudomonas aeruginosa*, showed an increasing trend over the past five years. Additionally, the study identified a significant temporal correlation between carbapenem usage and carbapenem resistance in Gram-negative bacteria, particularly in *Acinetobacter baumannii* and *Escherichia coli* (30). Similar to the previous study, consistent with these findings, our study revealed that MDRO VAP patients had higher and prolonged exposure to broad-spectrum antibiotics. The prolonged and frequent use of antibiotics in the ICU is one of the most significant factors contributing to the higher prevalence of MDR microorganisms. By limiting and shortening the use of broad-spectrum antimicrobials, antimicrobial stewardship programs can help reduce the development of antibiotic resistance, improve clinical outcomes, and lower healthcare costs (31).

Serum albumin and C-reactive protein (CRP) are widely recognized biomarkers for diagnosing and monitoring systemic inflammatory conditions, including pneumonia. The C-reactive protein-to-albumin ratio (CAR), a novel biomarker, was observed by Kunutsor et al. to be associated with an increased risk of pneumonia in middle-aged and older Finnish men (32). Our research also demonstrated significantly higher CAR levels in MDRO VAP patients, further confirming that hypoalbuminemia is an independent risk factor for MDRO VAP following cardiac valvular surgery. Albumin oxidation and breakdown affect the interactions with bioactive lipid mediators that are crucial for antimicrobial defense and tissue repair. A plausible biological mechanism suggests a causal relationship between hypoalbuminemia and an increased risk of both primary and secondary infections. Hypoalbuminemia is associated with the acquisition and severity of infectious diseases. Additionally, hypoalbuminemia can alter the pharmacokinetics and pharmacodynamics of antimicrobial drugs, thereby impacting their therapeutic effectiveness (33). Our findings indicated a significant decrease in preoperative albumin levels among patients with MDRO VAP, confirming that hypoalbuminemia is an independent risk factor for MDRO VAP after cardiac valvular surgery. A meta-analysis by Xu et al. demonstrated that hypoalbuminemia is significantly associated with increased mortality rates. Patients with hypoalbuminemia were more likely to experience postoperative complications, including infections, renal injury, pulmonary dysfunction, pneumonia, and other complications, compared to those with normal concentrations. Furthermore, hypoalbuminemia increased the risk of prolonged ICU stays, ventilatory support duration, and cardiopulmonary bypass time after cardiac surgery. Adjusting perioperative serum albumin levels and tailoring treatment strategies for high-risk patients may help reduce the incidence of complications, such as ventilator-associated pneumonia, and improve outcomes following heart valve surgery (34).

Additionally, we analyzed the prognosis of MDRO VAP patients. Patients with MDRO VAP had significantly longer durations of ICU stays, total hospital stays, and higher total hospitalization costs compared to those without MDRO VAP. Moreover, the MDRO VAP group exhibited an alarmingly high in-hospital mortality rate of 29.79%. A retrospective closed cohort Study also observed that the presence of multidrug-resistant pathogens in VAP patients is associated with a higher risk of death. The incidence of MDRO-VAP was 21.6%, and the mortality rate was 64.6% in critically ill patients undergoing mechanical ventilation. Patients with MDRO-VAP were less likely to be extubated and more likely to require a tracheostomy (35). These findings indicate that VAP caused by MDRO not only poses a significant challenge to clinical treatment but also increases risks and difficulties for patients and their families. Given the high morbidity and mortality associated with MDRO VAP, it is crucial to strictly implement prevention measures and treatment strategies for managing MDRO in VAP patients. More attention should be focused on patients who have risk factors associated with MDRO VAP infections.

This study has several limitations, including its retrospective, single-center design and small sample size. First, the small sample size may affect the generalizability and statistical power of the results. Second, the observational study design limits the ability to control for confounding factors, which could have influenced the results. Third, a retrospective evaluation of the impact of colonization with MDRO in patients with ventricular assist devices (VAD) found that MDRO colonization was associated with an increased risk of subsequent VAD infection (36). In this study, the correlation between preoperative MDRO colonization and postoperative MDRO infection was not analyzed; further research is needed to clarify the association between MDRO colonization and the subsequent development of MDRO infection after cardiac valvular surgery. Finally, in surgical patients with multiple sites of infection, it is challenging to distinguish between active infection and colonization in pulmonary isolates. This can affect the accuracy of diagnosing VAP and understanding its implications. These limitations highlight the need for cautious interpretation of the study's results and suggest that further multicenter, large-sample studies are recommended to validate the findings.

## 5 Conclusions

This study highlights that patients with VAP who received extended broad-spectrum antibiotic therapy, experienced prolonged periods of mechanical ventilation and had lower preoperative albumin levels were more likely to develop MDRO VAP following cardiac valvular surgery. Patients with MDRO VAP exhibited significantly worse outcomes, including longer ICU stays, extended total hospital stays, higher overall hospitalization costs, and increased in-hospital mortality rates. It is recommended that further multi-center, large-sample studies be conducted to validate the risk factors for MDRO VAP.

## Data Availability

The original contributions presented in the study are included in the article/Supplementary Material, further inquiries can be directed to the corresponding author.
